# Seroprevalence of hepatitis B and C viruses and some possible associated factors among cancer patients at the Oncology Treatment Center, Gondar, Northwest Ethiopia: A cross-sectional study

**DOI:** 10.1371/journal.pone.0308161

**Published:** 2024-08-02

**Authors:** Tadesse Atanaw, Getu Girmay, Aragaw Zemene, Muluneh Assefa, Tewodros Eshetie, Gezahegn Bewket, Fikadu Alemiye, Debaka Belete, Nega Birhane

**Affiliations:** 1 University of Gondar Comprehensive Specialized Hospital, Gondar, Ethiopia; 2 Department of Immunology and Molecular Biology, School of Biomedical and Laboratory Science, College of Medicine and Health Sciences, University of Gondar, Gondar, Ethiopia; 3 Department of Medical Biotechnology, Institute of Biotechnology, University of Gondar, Gondar, Ethiopia; 4 Department of Medical Microbiology, School of Biomedical and Laboratory Science, College of Medicine and Health Sciences, University of Gondar, Gondar, Ethiopia; 5 Department of Internal Medicine, School of Medicine, College of Medicine and Health Sciences, University of Gondar, Gondar, Ethiopia; PLOS ONE, UNITED KINGDOM OF GREAT BRITAIN AND NORTHERN IRELAND

## Abstract

**Background:**

Cancer patients are prone to infections such as hepatitis B virus (HBV) and hepatitis C virus (HCV), which pose a major public health challenge, especially in developing countries. However, little is known about the magnitude of these infections among cancer patients in Ethiopia. Thus, this study determined the prevalence of HBV and HCV in cancer patients at the Oncology Treatment Center, Gondar, Northwest Ethiopia.

**Materials and methods:**

An institutional-based cross-sectional study was conducted on 115 cancer patients from 15 April to 22 July 2023 at the Oncology Treatment Center, Gondar, Northwest Ethiopia. Sociodemographic, clinical, and other relevant data were collected using a pretested structured questionnaire. Five milliliters of venous blood were collected using a vacutainer tube, serum was harvested and tested for HBV and HCV using a one-step HBsAg and anti-HCV test strip with further confirmation through an ELISA test kit. Data were analyzed using SPSS version 20 and Fisher exact test was used to determine the association between HBV/HCV infection and associated factors.

**Results:**

Out of 115 cancer patients, the majority (62.6%) were females. The median age was 50 (IQR; 40–56) years. The overall prevalence of HBV and HCV infections was 4.3% (95% CI; 0.6–8%) and 6.1% (95% CI; 1.7–10.5%), respectively. Sex was significantly associated with the prevalence of HCV (p = 0.011) with higher anti-HCV positivity in males (14%) than in females (1.4%).

**Conclusions:**

In this study, the prevalence of HCV was higher and the HBV prevalence was intermediate in cancer patients. To reduce the burden of HBV and HCV infections, it is crucial to provide access to HBV and HCV screening services, strengthen vaccination, and improve prompt treatment in cancer patients.

## Introduction

Cancer is a large and heterogeneous group of malignant tumors that can affect any organ in the body and is characterized by the uncontrolled development and proliferation of cells [1]. Worldwide, it is the leading cause of morbidity and mortality with an anticipated 20 million new cases and 9.7 million deaths in 2022 [2, 3]. The impaired immune system in cancer patients along with the immunosuppressant therapy leads to the increase in the risk of viral infections in these patients. In developing countries, viral hepatitis and human papillomavirus (HPV) infections account for about 30% of cancer cases [4, 5].

Globally, an estimated 292 million individuals had a chronic HBV infection in 2016 and 71.1 million had a chronic HCV infection in 2015 [6]. The highest HCV distribution was reported in the WHO Eastern Mediterranean Region. While, HBV mostly affects the Western Pacific and the WHO African regions [7, 8]. The World Health Organization (WHO) reported three ranges of HBV infection prevalence as high (>8%), intermediate (2–7%), and low (< 2%). Similarly, the prevalence of HCV infection ranged from low (<1.5%), moderate (1.5–3.5%), and high (>3.5%) [9, 10]. In Africa, about 70 million individuals have chronic viral hepatitis, of which 60 million are caused by HBV infections and 10 million by HCV infections [11]. Besides, an Ethiopian meta-analysis study also reported a 7.4% and 3.1% pooled prevalence of HBV and HCV infections, respectively [12]. There has been a substantial increase in HBsAg seropositivity (14.4%) and anti-HCV seropositivity (12.4%) among clinically suspected hepatitis patients in Gondar [13].

Studies have shown a correlation between HBV and HCV and various cancer types other than hepatocellular carcinoma, leading to a rise in the number of new cases and the reactivation of latent infections [14–16]. Cancer patients have been found to have a higher prevalence of HBV and HCV infections [17–20]. Previously, there was a single study that reported the prevalence of HBV and HCV infection among women suspected of cervical cancer in the study area [21]. However, the previous study only focuses on cervical cancer suspected women and no reports on the prevalence of HBV/HCV among cancer patients in general in the study area. Therefore, the current study determined the prevalence of HBV and HCV infections among cancer patients at the Oncology Treatment Center, Gondar, Northwest Ethiopia.

## Materials and methods

### Study design, setting, and period

An institutional-based cross-sectional study was conducted among cancer patients at the University of Gondar Comprehensive Specialized Hospital (UoG-CSH) Oncology Treatment Center, Northwest Ethiopia from 15 April to 22 July 2023. The UoG-CSH is located in the Central Gondar Zone of the Amhara Regional state, 747 km from Addis Ababa (the capital city of Ethiopia). In Gondar town, there are 8 Health Centers and one Comprehensive Specialized Hospital (UoG-CSH). The UoG-CSH is a teaching hospital and offers a comprehensive specialty and referral service for more than seven million individuals in the catchment area. The UoG-CSH Oncology Treatment Center currently has 32 beds and serves more than 2500 cancer patients annually.

### Source and study populations

All cancer patients seeking service at the UoG-CSH Oncology Treatment Center were our source population. Cancer patients aged 18 years and above, both males and females, and confirmed for any type of cancer, and cancer patients who were both on treatment and treatment naïve who were available during the study period were our study populations. Cancer patients who were critically ill or extremely malnourished; and those who were in the intensive care unit (ICU) were not included in the study because those patients were unable to give blood samples.

### Sample size determination and sampling procedure

The sample size was calculated using a single population proportion formula, by considering the following assumptions: a 7.4% pooled prevalence of HBV obtained from an Ethiopian meta-analysis study [21] with a precision error of 5%, a confidence level of 95%, and considering 10% non-response rate. Then, the ultimate sample size obtained was 115 cancer patients. Systematic random sampling was used; data from the UoG-CSH Oncology Treatment Center revealed that around 7 cancer patients visited per day. Thus, the estimated population (N) during the study period was (N = 7 cancer patients per day* 5 working days per week* 4 weeks per month* 4 months of study period = 560 cancer patients). To calculate the interval (k) value, divide the estimated population (N) by sample size (n), which was 560/115 = 5. We included the first study participant at random and consecutively recruited every 5^th^ cancer patient who visited the UoG-CSH Oncology Treatment Center from April to July 2023, until the ultimate sample size was attained.

### Data collection

Sociodemographic, clinical, and other relevant data were collected using a pretested structured questionnaire through a well-trained clinical nurse who worked in the UoG-CSH Oncology Treatment Center (**S1 Table**).

### Blood sample collection and processing

Five milliliters (5ml) of venous blood was obtained using a serum separator tube (SST) by an experienced laboratory technician. Blood samples were transported to the UoG-CSH serology laboratory and the blood was left to clot at room temperature without any disturbances for 30 minutes. Then, serum was harvested through centrifugation for 15 minutes at 5000 revolutions per minute (RPM). The serum was stored at -20°c until tested for HBsAg and anti-HCV.

### HBsAg and anti-HCV testing

The HBsAg and anti-HCV antibody test was performed using a one-step HBsAg test strip (Guangzhou Wondfo Biotech Co., Ltd., China) and one step anti-HCV test strip (Hangzhou Alltest Biotech Co., Ltd., China), respectively as per the manufacturer’s instruction. For HBsAg detection, the kits’ sensitivity and specificity were 96.2% and 99.3%, respectively, and for anti-HCV detection, they were 98.7% and 99.1%, respectively [22, 23]. The samples that tested positive for HBsAg and anti-HCV were further confirmed through the commercial Enzyme-Linked Immunosorbent Assay (ELISA) test kit (AiD^TM^ HBsAg and anti-HCV antibody, Beijing Wantai Biological Pharmacy Enterprise Co., Ltd., China) following the manufacturer’s instruction [24, 25]. The procedure for collecting and processing samples was carried out following the national and international laboratory protocols for handling, processing, and collection of biological specimens [26, 27].

### Data management and quality control

Before data collection, the questionnaire underwent a pretest with a 5% sample to ensure clarity, and any amendments that were required have been implemented based on the outcomes. The primary investigator provided training to the data collectors and supervised them during the data collection period. A trained and experienced laboratory technician collected the blood samples, and standard operating protocols were followed for sample collection, processing, and laboratory testing. The samples tested positive for HBsAg and anti-HCV had tested in duplicate and further confirmed using the commercial ELISA test kit. All test results were positive using HBsAg and Anti-HCV test strips were also positive for the ELISA test kit. Along the test kits, positive and negative control serum samples were tested to ensure the reliability and precision of the results.

### Data analysis

Data were entered, cleaned, and analyzed using SPSS version 20 software. Descriptive statistics such as; proportions, measures of central tendency, and cross-tabulations were computed to characterize study participants and to determine the prevalence of HBV and HCV infections. Fisher exact test was used to observe the association between associated factors and the prevalence of HBV and HCV infections. A p-value of ≤ 0.05, with a 95% confidence interval was considered statistically significant.

### Ethical statement

Ethical approval was obtained from the ethics committee of the Institute of Biotechnology, University of Gondar, Gondar, Ethiopia, with a protocol number; IOB/136/04/2023. All study participants provided written informed consent and a support letter was obtained from the UoG-CSH medical director. Each study participant was assigned a unique code to ensure their confidentiality, and the study was conducted as per the Helsinki Declaration for Biomedical Research [28].

## Results

### Socio-demographic and clinical characteristics of study participants

A total of 115 cancer patients were included in this study. Out of them, 72 (62.6%) were females and 43 (37.4%) were males. The median (IQR) age was 50 (40–56) years. The majority of cancer patients (52.2%) were within the 36–50 years of age range followed by 51–65 years of age (23.5%). Rural inhabitants made up sixty-two (53.9%) of cancer patients. Among cancer patients, 31 (27%) had breast cancer, followed by 20 (17.4%) with blood cancer and 20 (17.4%) with gastrointestinal cancer (**Table 1**). Thirty-three (27.8%) of cancer patients were newly diagnosed with ungrouped cancer stage and 27 (23.5%) had stage four cancer. Of all cancer patients, during the study period; 8.7% (10/115) had multiple infections and the co-morbidities were diabetes (2.5%), HIV (1.8%), hypertension (1.8%), tuberculosis (0.9%), and GI and liver infection (1.8%) (**Table 1**).

**Table 1:** Sociodemographic and clinical characteristics of cancer patients at the UoG-CSH Oncology Treatment Center, Northwest Ethiopia.

Variables	Category	Frequency (n)	Percentage (%)
Sex	Male	43	37.4
	Female	72	62.6
Age (Years)	20–35	11	9.6
	36–50	60	52.2
	51–65	27	23.5
	> 65	17	14.8
Residence	Urban	53	46.1
	Rural	62	53.9
Educational level	No formal education	38	33.0
	Primary	34	29.6
	Secondary	30	26.1
	College and above	13	11.3
Occupational status	Government-employed	11	9.6
	Self-employed	32	27.8
	Farmer	44	38.3
	Others	28	24.3
Marital status	Single	14	12.2
	Married	85	73.9
	Divorced	10	8.7
	Widowed	6	5.2
Sign of jaundice	Yes	15	13.0
	No	100	87.0
Types of cancer	Blood cancer	20	17.4
	Breast cancer	31	27.0
	Urinary tract cancer	15	13.1
	Gastrointestinal cancer	20	17.4
	Liver cancer	8	6.9
	Respiratory cancer	11	9.6
	Thyroid, pancreatic, and spleen cancer	5	4.3
	Squamous, soft tissue sarcoma, and bone cancer	5	4.3
Cancer stage	Ungrouped/newly diagnosed	33	28.7
	I	6	5.2
	II	24	20.9
	III	25	21.7
	IV	27	23.5
Co-morbidities	No co-morbidity	105	91.3
	Diabetes	3	2.6
	HIV	2	1.7
	Tuberculosis	1	0.9
	Hypertension	2	1.7
	GI and Liver infections	2	1.7
Lymphocyte count (median ± IQR)	25 (14–42)	-	-
WBC count (median ± IQR)	5300 (3700–7650)	-	-

IQR: interquartile range; WBC: White blood cell; HIV: Human immunodeficiency virus; GI; Gastrointestinal.

### Seroprevalence of HBV and HCV infections

Overall, 4.3% (95% CI; 0.6–8) and 6.1% (95% CI; 1.7–10.5) of cancer patients had HBV and HCV, respectively. The proportion of HBV infection was higher in males (9.3%) compared to females (1.4%) and HBV prevalence was 6.7% among the 36–50 years age group. Cancer patients who attended primary education and those with no formal education had HBsAg positivity rates of 11.8% and 2.6%, respectively. Among cancer patients who had married; 5.9% (5/85) of them were positive for HBsAg. From cancer patients who had liver cancer, urinary tract cancer, blood cancer, and gastrointestinal cancer; 25%, 6.7%, 5%, and 5% of them were positive for HBsAg (**Table 2**).

**Table 2:** Distribution of HBsAg and anti-HCV seropositivity with sociodemographic and clinical characteristics.

Variables	Category	HBV status (N = 115)		HCV status (N = 115)	
		Positive (%)	Negative (%)	Positive (%)	Negative (%)
Sex	Male	4 (9.3)	39 (90.7)	6 (14)	37 (86)
	Female	1 (1.4)	71 (98.6)	1 (1.4)	71 (98.6)
Age (Years)	20–35	0 (0)	11 (100)	0 (0)	11 (100)
	36–50	4 (6.7)	56 (93.3)	5 (8.3)	55 (91.7)
	51–65	1 (3.7)	26 (96.3)	2 (7.4)	25 (92.6)
	> 65	0 (0)	17 (100)	0 (0)	17 (100)
Residence	Urban	3 (5.9)	48 (94.1)	5 (9.8)	46 (90.2)
	Rural	2 (3.1)	62 (96.9)	2 (3.1)	62 (96.9)
Educational level	No formal education	1 (2.6)	37 (97.4)	2 (5.3)	36 (94.7)
	Primary	4 (11.8)	30 (88.2)	1 (2.9)	33 (97.1)
	Secondary	0 (0)	30 (100)	3 (10)	27 (90)
	College and above	0 (0)	13 (100)	1 (7.7)	12 (92.3)
Occupational status	Government-employed	0 (0)	11 (100)	1 (9.1)	10 (90.9)
	Self-employed	2 (6.3)	30 (93.7)	0 (0)	32 (100)
	Farmer	1 (2.3)	43 (97.7)	3 (6.8)	41 (93.2)
	Others	2 (7.1)	26 (92.9)	3 (10.7)	25 (89.3)
Marital status	Single	0 (0)	14 (100)	3 (21.4)	11 (78.6)
	Married	5 (5.9)	80 (94.1)	4 (4.7)	81 (95.3)
	Divorced	0 (0)	10 (100)	0 (0)	10 (100)
	Widowed	0 (0)	6 (100)	0 (0)	6 (100)
Sign of jaundice	Yes	2 (13.3)	13 (86.7)	2 (13.3)	13 (86.7)
	No	3 (3)	97 (97)	5 (5)	95 (95)
Types of cancer	Blood cancer	1 (5)	19 (95)	2 (10)	18 (90)
	Breast cancer	0 (0)	31 100)	1 (3.2)	30 (96.8)
	Urinary tract cancer	1 (6.7)	14 (93.3)	0 (0)	15 (100)
	Gastro-intestinal cancer	1 (5)	19 (95)	2 (10)	18 (90)
	Liver cancer	2 (25)	6 (75)	0 (0)	8 (100)
	Respiratory cancer	0 (0)	11 (100)	2 (18.2)	9 (81.8)
	Thyroid, pancreatic, and spleen cancer	0 (0)	5 (100)	0 (0)	5 (100)
	Squamous, soft tissue sarcoma, and bone cancer	0 (0)	5 (100)	0 (0)	5 (100)
Cancer stage	Ungrouped	2 (6.1)	31 (93.9)	4 (12.1)	29 (87.9)
	I	0 (0)	6 (100)	1 (16.7)	5 (83.3)
	II	1 (4.2)	23 (95.8)	1 (4.2)	23 (95.8)
	III	0 (0)	25 (100)	1 (4)	24 (96)
	IV	2 (7.4)	25 (92.6)	0 (0)	27 (100)
Co-morbidities	No co-morbidity	4 (3.8)	101 (87.2)	7 (6.7)	98 (93.3)
	Diabetes	0 (0)	3 (100)	0 (0)	3 (100)
	HIV	1 (50)	1 (50)	0 (0)	2 (100)
	Tuberculosis	0 (0)	1 (100)	0 (0)	1 (100)
	Hypertension	0 (0)	2 (100)	0 (0)	2 (100)
	GI and Liver infections	0 (0)	2 (100)	0 (0)	2 (100)

HIV: Human immunodeficiency virus; HBV: Hepatitis B virus; HCV: Hepatitis C virus.

Likewise, male cancer patients (14%) had a higher prevalence of anti-HCV positivity compared to females (1.4%). Cancer patients in the 36–50 and 51–65 years age group had an anti-HCV positivity rate of 8.3% and 7.4%, respectively. Higher HCV prevalence was observed in rural dwellers (9.8%) compared to urban residents (3.1%). Cancer patients with secondary education, no formal education, and primary education showed HCV prevalence of 10%, 5.3%, and 2.9%, respectively. From patients with respiratory tract cancer (18.2%), gastrointestinal cancer (10%), breast cancer (3.2%), and blood cancer (10%) were tested positive for anti-HCV (**Table 2**).

### Seroprevalence of HBV and HCV infections and associated factors

In this study, we applied the Fisher exact test to demonstrate the relationship between HBsAg and anti-HCV seropositivity and some possible associated factors. Cancer patients who are within the 36–50 years of age range (6.7%) and males (9.3%) had a higher prevalence of HBsAg positivity. However, there was no significant association of HBsAg positivity with age (p = 0.898) and sex (p = 0.064). Cancer patients who attended primary education (11.8%) had higher HBsAg positivity; but, the association was not statistically significant (p = 0.115). Among cancer patients who had a history of multiple sexual partners, a contact history of HBV, and a history of surgery; 7.7%, 6.3%, and 3.6% of them had tested positive for HBsAg. Nevertheless, there was no significant association between any of these associated factors and HBsAg positivity (**Table 3**).

**Table 3:** Seropositivity of HBsAg and its associated factors among cancer patients.

Variables	Category	HBV status (N = 115)		
		Positive (%)	Negative (%)	*P* value
Age (Years)	20–35	0 (0)	11 (100)	0.898
	36–50	4 (6.7)	56 (93.3)	
	51–65	1 (3.7)	26 (96.3)	
	> 65	0 (0)	17 (100)	
Sex	Male	4 (9.3)	39 (90.7)	0.064
	Female	1 (1.4)	71 (98.6)	
Educational level	No formal education	1 (2.6)	37 (97.4)	0.115
	Primary	4 (11.8)	30 (88.2)	
	Secondary	0 (0)	30 (100)	
	College and above	0 (0)	13 (100)	
Contact history of HBV	Yes	1 (6.3)	15 (93.7)	0.534
	No	4 (4)	95 (96)	
History of STI	Yes	0 (0)	25 (100)	0.584
	No	5 (5.6)	85 (94.4)	
Multiple sexual partner	Yes	1 (7.7)	12 (92.3)	0.457
	No	4 (3.9)	98 (96.1)	
Blood transfusion history	Yes	1 (3.1)	31 (96.9)	1.000
	No	4 (4.8)	79 (95.2)	
Tattooing	Yes	0 (0)	14 (100)	1.000
	No	5 (4.3)	110 (95.7)	
History of surgery	Yes	1 (3.6)	27 (96.4)	1.000
	No	4 (4.6)	83 (95.4)	
Sharing sharp materials	Yes	1 (2.4)	41 (97.6)	0.651
	No	4 (5.5)	69 (94.5)	

STI: sexually transmitted infection; HBV: Hepatitis B virus.

Anti-HCV positivity and sex were significantly associated (p = 0.011), and male cancer patients had higher anti-HCV positivity (14%) compared to females (1.4%). The 36 to 50-year-old age group had a higher percentage of anti-HCV positivity (8.3%), although there was no significant association between age and the HCV prevalence (p = 0.766). Among cancer patients who had a history of multiple sexual partners, prior surgery, sharing sharp materials, and sexually transmitted infection (STI) exposure; 15.4%, 10.7%, 9.5%, and 8% of them had tested positive for anti-HCV, respectively. However, no significant association was found between any of these associated factors and the prevalence of HCV infection (**Table 4**).

**Table 4:** Seropositivity of anti-HCV and its associated factors among cancer patients.

Variables	Category	HCV status (N = 115)		
		Positive (%)	Negative (%)	*P* value
Age (Years)	20–35	0 (0)	11 (100)	0.766
	36–50	5 (8.3)	55 (91.7)	
	51–65	2 (7.4)	25 (92.6)	
	> 65	0 (0)	17 (100)	
Sex	Male	6 (14%)	37 (86)	0.011
	Female	1 (1.4)	71 (98.6)	
Educational level	No formal education	2 (5.3)	36 (94.7)	0.644
	Primary	1 (2.9)	33 (97.1)	
	Secondary	3 (10)	27 (90)	
	College and above	1 (7.7)	12 (92.3)	
Contact history of HCV	Yes	0 (0)	16 (100)	0.591
	No	7 (7.1)	92 (92.9)	
History of STI	Yes	2 (8)	23 (92)	0.645
	No	5 (5.6)	85 (94.4)	
Multiple sexual partner	Yes	2 (15.4)	11 (84.6)	0.179
	No	5 (4.9)	97 (95.1)	
Blood transfusion history	Yes	2 (6.3)	30 (93.7)	1.000
	No	5 (6)	78 (94)	
History of surgery	Yes	3 (10.7)	25 (89.3)	0.359
	No	4 (4.6)	83 (95.4)	
Sharing sharp material	Yes	4 (9.5)	38 (90.5)	0.256
	No	3 (4.1)	70 (95.9)	

STI: sexually transmitted infection; HCV: Hepatitis C virus.

## Discussion

In the present study, we investigated the prevalence of HBV and HCV infections in cancer patients attending the Oncology Treatment Center at the UoG-CSH, Northwest Ethiopia. Infections with HBV and HCV pose a significant public health challenge, especially in low- and middle-income countries [29, 30]. More than 70 million Africans have chronic viral hepatitis, of which 60 million are caused by HBV and 10 million by HCV [11].

The higher burden of HBV and HCV infections in contexts with limited resources has been linked to several factors. Among them are insufficient preventative actions (such as immunization) and inadequate treatment and care for the infected individuals [31, 32]. Studies suggested that approximately 25% of HCV-infected cancer patients receiving chemotherapy undergo HCV reactivation, while patients taking chemotherapy for cancer are most likely to have HBV reactivation [33, 34].

In this study, we found an overall prevalence of 4.3% and 6.1% for HBV and HCV infections, respectively (**Table 2**). Based on the WHO classification criteria [9], the current prevalence of HCV infection was higher and the HBV prevalence was intermediate. The current HBV prevalence was comparable with previous findings from Gondar among cervical cancer suspected women; 2.5% [21] and HBsAg prevalence studies conducted in Ethiopia, where the HBV prevalence rate ranged from 3% to 8% [35–40]. Furthermore, our findings were also comparable with studies from Egypt; 3.6% [41], Turkey; 2.4% [42], 3.4% [43], and 4.2% [44], Cameroon; 7.7% [45], China; 6.3% [46], Taiwan; 7.78% [47], and the USA; 6.5% [48]. On the other hand, the current HBV prevalence was lower compared to findings from Gondar; 14.4% [13], Debre Tabor; 13% [49], and Addis Ababa; 10.8% [20]. The current HBV prevalence was also found to be lower compared to study findings among different Oncology patients from Nigeria [19, 50], Kenya [18], Sudan [51], Zimbabwe [17], and France [52], where the HBV prevalence was ranged from 8.5% to 48.3%. These discrepancies might arise from differences in sample size, study population, geographic variation, and variation in HBV endemicity and risk behavior.

The HCV prevalence in our study was consistent with previous findings from Bahir Dar, Ethiopia among participants who were booked for surgery procedures (4.3%) [40]. Moreover, the current finding was also in line with study findings from Nigeria [19], Burkina Faso [53], Cameroon [45], and the USA [48], where the HCV prevalence reported was 2%, 2.3%, 2.2%, and 2.4%, respectively. However, our finding was higher than several studies conducted in Ethiopia [20, 21, 35, 36], where the HCV prevalence ranged from 0.7% to 1.7%. This variation might be due to differences in the study population and differences in the diagnostic methods; we applied one-step HBsAg and anti-HCV test strips with ELISA as a confirmatory test but some of the previous studies used only rapid immunochromatographic test kits.

Moreover, the present HCV prevalence was found to be higher than previous findings on different cancer patients from Kenya; 1.2% [18], Egypt; 0.9% [41], Turkey; 0.7% [44], China; 1.2% [46], and France; 1.3% [52]. The observed discrepancies might be attributed to geographic variation, differences in the risk of acquiring HCV, and the impact of educational level. Particularly in our study, most participants had no formal education; which could contribute to the higher burden of HCV due to inadequate screening services, insufficient community awareness, and limited access to medical facilities. On the other hand, the current HCV prevalence was lower compared to study findings from Gondar [13], Zimbabwe [17], Pakistan [54], and Turkey [42], where the HCV prevalence was reported; 12.4%, 20%, 18.33%, and 28.2%, respectively. Those variations might be due to differences in the study population, socioeconomic status of the study participants, and variations in the HCV risk behavior.

In this study, male cancer patients and participants aged 36 to 50 years had a higher prevalence of HBsAg and anti-HCV positivity (**Tables 3** and **4**). The Fisher exact test showed that sex was significantly associated with HCV prevalence, but not age. This finding was supported by previous investigations [19, 20, 45, 50]. This might have been the result of hormonal differences, cultural variation, and physical characteristics which render men more vulnerable to HCV infections. Although there were no significant associations between the prevalence of HBV and HCV with the associated factors assessed in this study; from cancer patients who had a history of sharing sharp materials, previous surgery, STI exposure, and multiple sexual partners 9.5%, 10.7%, 8%, and 15.4% of them had tested positive for anti-HCV. Those findings were supported through previous studies [21, 40, 51]. Since sharing sharp materials, unsafe sexual relations, and blood contact are the major ways of HCV transmission, these variables could contribute to the higher prevalence of HCV in cancer patients [55]. As a limitation of the study, the temporal relationship between HBV or HCV seropositivity and cancer infection, the seroprevalence of HBV or HCV infection, and associated factors could not be addressed due to the cross-sectional nature of the study. In addition, we did not compute the bivariate and multivariate logistic regression analyses to observe the strength of the association between the prevalence of HBV and HCV infections and associated factors due to the small number of positive cases in each category of associated factors.

## Conclusion and recommendations

As per the WHO (1990) viral hepatitis B and C endemicity classification criteria [9]. We found a higher prevalence of HCV and an intermediate prevalence of HBV infection in cancer patients. A higher prevalence of anti-HCV positivity was observed in men and the age range of 36–50 years. Sex was found to be significantly associated with the prevalence of HCV infections. To reduce the burden of HBV and HCV infections in cancer patients, it is crucial to increase public awareness, provide access to HBV and HCV screening services, improve prompt treatment of HCV and HBV infection, and strengthen infection prevention methods (such as; HBV vaccination).

## Supporting information

S1 Checklist STROBE checklist for observational study. (DOCX)

S1 Table Sociodemographic, clinical, and associated factor assessment questionnaire. (PDF)

S1 Dataset Data set used for analysis. (SAV)

## Abbreviations

Anti-HCV: Antibody to Hepatitis C Virus
ELISA: Enzyme-linked immunosorbent assay
HBsAg: Hepatitis B surface antigen
HBV: Hepatitis B Virus
HCV: Hepatitis C Virus
STI: Sexually transmitted infection
UoG-CSH: University of Gondar Comprehensive Specialized Hospital
WHO: World Health Organization
