# Supplementary material for: Seroprevalence of hepatitis B and C viruses and some possible associated factors among cancer patients at the Oncology Treatment Center, Gondar, Northwest Ethiopia: A cross-sectional study
Source: PLoS One. 2024 Aug 2;19(8):e0308161. doi: 10.1371/journal.pone.0308161 (PMC11296633; doi:10.1371/journal.pone.0308161)
Supplement: S1 Checklist — (DOCX) [file pone.0308161.s001.docx]

S1 File: STROBE checklist for observational study

|  | Item No. | Recommendation | Page No | Relevant text from manuscript |
| --- | --- | --- | --- | --- |
| **Title and abstract** | 1 | (a) Indicate the study’s design with a commonly used term in the title or the abstract | 1 | A Hospital based cross-sectional study |
|  |  | (b) Provide in the abstract an informative and balanced summary of what was done and what was found | 1-2 |  |
| Introduction | | | |  |
| Background/rationale | 2 | Explain the scientific background and rationale for the investigation being reported | 3-4 |  |
| Objectives | 3 | State specific objectives, including any prespecified hypotheses | 4 |  |
| Methods | | | |  |
| Study design | 4 | Present key elements of study design early in the paper | 4 |  |
| Setting | 5 | Describe the setting, locations, and relevant dates, including periods of recruitment, exposure, follow-up, and data collection | 4 | Cancer patients at the University of Gondar Comprehensive Specialized Hospital Oncology Treatment Center from April to July 2023 |
| Participants | 6 | Cross-sectional study—Give the eligibility criteria, and the sources and methods of selection of participants | 4-5 |  |
| Variables | 7 | Clearly define all outcomes, exposures, predictors, potential confounders, and effect modifiers. Give diagnostic criteria, if applicable |  | NA |
| Data sources/ measurement | 8* | For each variable of interest, give sources of data and details of methods of assessment (measurement). | 5-6 | Serum samples were harvested from 5 mL of venous blood; HBV and HCV prevalence was determined using a one-step HBsAg and anti-HCV test strip with further confirmation through the ELISA test kit. |
| Bias | 9 | Describe any efforts to address potential sources of bias |  | NA |
| Study size | 10 | Explain how the study size was arrived at | 5 | The sample size was calculated using a single population proportion, by considering: A 7.4% HBV prevalence, 5% precision error, 95% CL, and Describe comparability of assessment methods if there is more than one group 10% non-response rate. Gives an ultimate sample size of 115. |

| Quantitative variables | 11 | Explain how quantitative variables were handled in the analyses. If applicable, describe which groupings were chosen and why | 5-6 |  |
| --- | --- | --- | --- | --- |
| Statistical methods | 12 | (a) Describe all statistical methods, including those used to control for confounding | 7 |  |
|  |  | (b) Describe any methods used to examine subgroups and interactions | 7 |  |
|  |  | (c) Explain how missing data were addressed | 7 |  |
|  |  | (d) Cross-sectional study—If applicable, describe analytical methods taking account of sampling strategy | 7 | . |
|  |  | (e) Describe any sensitivity analyses |  | NA |
| Results | | | | |
| Participants | 13* | (a) Report numbers of individuals at each stage of study—eg numbers potentially eligible, examined for eligibility, confirmed eligible, included in the study, completing follow-up, and analysed | 8 | A total of 115 cancer patients were included in this study. Out of them, 72 (62.6%) were females and 43 (37.4%) were males. The median (IQR) age was 50 (40-56). |
|  |  | (b) Give reasons for non-participation at each stage |  | NA |
|  |  | (c) Consider use of a flow diagram |  | NA |
| Descriptive data | 14* | (a) Give characteristics of study participants (eg demographic, clinical, social) and information on exposures and potential confounders | 8-9 |  |
|  |  | (b) Indicate number of participants with missing data for each variable of interest |  | NA |
| Outcome data | 15* | Cross-sectional study—Report numbers of outcome events or summary measures | 9-10 |  |
| Main results | 16 | (a) Give unadjusted estimates and, if applicable, confounder-adjusted estimates and their precision (eg, 95% confidence interval). Make clear which confounders were adjusted for and why they were included | 10-15 |  |
|  |  | (b) Report category boundaries when continuous variables were categorized |  | NA |
|  |  | (c) If relevant, consider translating estimates of relative risk into absolute risk for a meaningful time period |  | NA |

Continued on next page

| Other analyses | 17 | Report other analyses done—eg analyses of subgroups and interactions, and sensitivity analyses |  | NA |
| --- | --- | --- | --- | --- |
| Discussion | | | | |
| Key results | 18 | Summarise key results with reference to study objectives | 16 | In this study, we found an overall prevalence of 4.3% (95% CI; 3.4 to 5.2) and 6.1% (95% CI; 5.2 to 7.0) for HBV and HCV infections, respectively |
| Limitations | 19 | Discuss limitations of the study, taking into account sources of potential bias or imprecision. Discuss both direction and magnitude of any potential bias | 18 | We did not compute the bivariate and multivariate logistic regression analyses to observe the strength of the association between risk factors and the prevalence of HBV and HCV infections due to the limited sample size in each category of associated risk factors |
| Interpretation | 20 | Give a cautious overall interpretation of results considering objectives, limitations, multiplicity of analyses, results from similar studies, and other relevant evidence | 15-18 |  |
| Generalisability | 21 | Discuss the generalisability (external validity) of the study results | 18 |  |
| Other information | |  | | |
| Funding | 22 | Give the source of funding and the role of the funders for the present study and, if applicable, for the original study on which the present article is based | 19 |  |
