# Supplementary material for: Seroprevalence of hepatitis B and C viruses and some possible associated factors among cancer patients at the Oncology Treatment Center, Gondar, Northwest Ethiopia: A cross-sectional study
Source: PLoS One. 2024 Aug 2;19(8):e0308161. doi: 10.1371/journal.pone.0308161 (PMC11296633; doi:10.1371/journal.pone.0308161)
Supplement: S1 Table — (PDF) [file pone.0308161.s002.pdf]

**S 1 Table: Sociodemographic, clinical, and associated factor assessment questionnaire.**

| No | Variables | Response |
|---|---|---|
| **A. Sociodemographic characteristics** | | |
| 101 | Age | _________ years |
| 102 | Sex | 1. Male 2. Female |
| 103 | Residence | 1. Urban 2. Rural |
| 104 | Educational level | 1. No formal education<br>2. Primary Education<br>3. Secondary Education<br>4. College and above |
| 105 | Occupational status | 1. Government employed<br>2. Self-employed<br>3. Farmer<br>4. Others; specify________ |
| 106 | Marital status | 1. Single<br>2. Married<br>3. Divorced<br>4. Widowed |
| **B. Clinical Characteristics** | | |
| 201 | Sign of jaundice | 1. Yes 2. No |
| 202 | Types of Cancer | __________________ |
| 203 | Stage of cancer | 1. Ungrouped/newly diagnosed<br>2. Stage I 3. Stage II 4. Stage III 5. Stage IV |
| 204 | Comorbidity/Co-infection | _______________ |

| | | |
|---|---|---|
| 205 | Total white blood cell count | _______________ per $10^3$ cells/µl |
| 206 | Total Lymphocyte count | _______________ per $10^3$ cells/µl |
| **C. Assessing associated factors for HBV and HCV infections** | | |
| 301 | Age in years | ___________ |
| 302 | Sex | 1. Male 2. Female |
| 303 | Educational level | 1. No formal education<br>2. Primary Education<br>3. Secondary Education<br>4. College and above |
| 304 | Contact history of HBV | 1. Yes 2. No |
| 305 | Contact history of HCV | 1. Yes 2. No |
| 306 | History of sexually transmitted infection (STI) | 1. Yes 2. No |
| 307 | Multiple sexual partner | 1. Yes 2. No |
| 308 | Previous blood transfusion history | 1. Yes 2. No |
| 309 | Tattooing | 1. Yes 2. No |
| 310 | History of previous surgery | 1. Yes 2. No |
| 311 | Practice on sharing sharp materials | 1. Yes 2. No |
